# Large‐scale plasma proteomics can reveal distinct endotypes in chronic obstructive pulmonary disease and severe asthma

**DOI:** 10.1002/clt2.12091

**Published:** 2021-12-20

**Authors:** Masaru Suzuki, John J. Cole, Satoshi Konno, Hironi Makita, Hiroki Kimura, Masaharu Nishimura, Rose A. Maciewicz

**Affiliations:** ^1^ Department of Respiratory Medicine, Faculty of Medicine and Graduate School of Medicine Hokkaido University Sapporo Japan; ^2^ GLAZgo Discovery Centre University of Glasgow Glasgow UK; ^3^ Hokkaido Medical Research Institute for Respiratory Diseases Sapporo Japan; ^4^ Respiratory, Inflammation and Autoimmunity, Innovative Medicines and Early Development Biotech Unit AstraZeneca Gothenburg Sweden

**Keywords:** COPD, endotypes, exosome, proteomics, severe asthma, BPCO, endotypes, exosomes, protéomique, asthme sévère

## Abstract

**Background:**

Chronic airway diseases including chronic obstructive pulmonary disease (COPD) and asthma are heterogenous in nature and endotypes within are underpinned by complex biology. This study aimed to investigate the utility of proteomic profiling of plasma combined with bioinformatic mining, and to define molecular endotypes and expand our knowledge of the underlying biology in chronic respiratory diseases.

**Methods:**

The plasma proteome was evaluated using an aptamer‐based affinity proteomics platform (SOMAscan®), representing 1238 proteins in 34 subjects with stable COPD and 51 subjects with stable but severe asthma. For each disease, we evaluated a range of clinical/demographic characteristics including bronchodilator reversibility, blood eosinophilia levels, and smoking history. We applied modified bioinformatic approaches used in the evaluation of RNA transcriptomics.

**Results:**

Subjects with COPD and severe asthma were distinguished from each other by 365 different protein abundancies, with differential pathway networks and upstream modulators. Furthermore, molecular endotypes within each disease could be defined. The protein groups that defined these endotypes had both known and novel biology including groups significantly enriched in exosomal markers derived from immune/inflammatory cells. Finally, we observed associations to clinical characteristics that previously have been under‐explored.

**Conclusion:**

This investigational study evaluating the plasma proteome in clinically‐phenotyped subjects with chronic airway diseases provides support that such a method can be used to define molecular endotypes and pathobiological mechanisms that underpins these endotypes. It provided new concepts about the complexity of molecular pathways that define these diseases. In the longer term, such information will help to refine treatment options for defined groups.

## 1 INTRODUCTION

Chronic airway diseases such as chronic obstructive pulmonary disease (COPD) and asthma are common and significant causes of morbidity and mortality. COPD is characterized by persistent respiratory symptoms and airflow limitation due to airway and alveolar abnormalities.^1^ Asthma is characterized by variable respiratory symptoms and expiratory airflow limitation.^2^ The differential diagnosis of these is problematic especially in older adults, due to the presence of overlapping clinical features. For instance, fixed airflow limitation is observed in patients with severe asthma^3^, ^4^ with distinct phenotypes observed in smokers.^4^ While 50% of COPD patients had at least one asthma‐like feature (bronchodilator reversibility, blood eosinophilia, or atopy) even if they were not clinically diagnosed with asthma.^5^ Hidden in these clinical groups may be a diverse range of molecular endotypes, where lack of knowledge of the underlying pathobiology hampers determining the best treatment regime.

Study of biological networks that govern chronic airway diseases may help to identify the unique underlying biology. This concept of molecular endotypes in asthma was initially discussed in terms of type 2 and non‐type 2 asthma.^6^, ^7^ Extension to these endotypes have been proposed with the transcriptome analysis of peripheral blood,^8^ epithelial brushings and bronchial biopsies,^9^ as well as metabolomics.^10^ For COPD, a meta‐analysis of endotypes was achieved from peripheral blood gene expression analysis.^11^

However, as proteins are central to almost all cellular processes, and dysregulation of expression and function is associated with a range of disorders, it makes sense to assess proteome‐derived endotypes. In respiratory diseases, the de novo detection of such proteins has been limited to low throughput analysis usually of inflammatory mediators. The application of proteomics in clinical and research applications in respiratory disease has been recently reviewed^12^ including the developments in protein detection technologies, that enables the simultaneous quantitation of large numbers of circulating proteins, including low‐abundance analytes, with high sensitivity and precision cohorts.^13^, ^14^ The use of these has led to the identification of biomarkers signatures and new concepts about disease pathology in allergic skin disease,^15^ in respiratory disease such as idiopathic pulmonary fibrosis^16^ and bronchiectasis in cystic fibrosis,^17^ and in chronic diseases such as cardiovascular disease^18^ and inflammatory bowel disease.^19^

We hypothesized that molecular endotypes of COPD and severe asthma may be achieved through evaluation of the plasma proteome. Furthermore, we addressed whether using bioinformatics approaches adopted from the study of RNA sequencing data could help to elucidate the underlying biology. We evaluated the abundance of 1238 proteins in a subset of individuals from the Hokkaido COPD cohort^5^, ^20^, ^21^ and the Hokkaido‐based Investigative Cohort Analysis for Refractory Asthma (Hi‐CARAT)^4^, ^22^, ^23^ studies. Our results indicate that large scale plasma proteome approach offers potential to define novel molecular endotypes and unique underlying biology.

## 2 METHODS

Details of the methods are shown in the Supporting Information.

### 2.1 Patients cohorts

The protocols for the Hokkaido COPD cohort, Hi‐CARAT, and this study were approved by the ethics committee of Hokkaido University School of Medicine (med02‐001) and Hokkaido University Hospital (009‐0025, 015‐0336), respectively. They were performed in accordance with the Declaration of Helsinki. All subjects provided written, informed consent with an additional opt‐out consent for this study.

### 2.2 COPD cohort

A subset of Hokkaido COPD cohort subjects^5^, ^20^, ^21^ was selected (Figure S1). Subjects with physician‐diagnosis of asthma were excluded. To ensure we evaluated the broadest range of clinical features, we included those with asthma‐like features (*n* = 17): high blood eosinophil levels (>300/μl) and bronchodilator reversibility (ΔFEV_1_ ≥ 200 ml and ≥12% after inhalation of 400 μg of salbutamol, the average value for three visits taken during the first year) as well as without them (*n* = 17). Subject's baseline clinical measures were found to be stable as assessed by yearly evaluation of blood eosinophil levels and bronchodilator reversibility over 5 years. Sex, age, pack‐years, and BMI on this cohort can be found in the supplementary information datasets (Dataset‐1).

### 2.3 Asthma cohort

A subset of Hi‐CARAT severe asthmatics^4^, ^22^, ^23^ (hereafter “asthma”) was selected (Figure S1). To ensure we evaluated the broadest range of clinical features, we included those with smoking history (≥10 pack‐years) and low (<150/μl) (*n* = 17) or high (>300/μl) (*n* = 17) blood eosinophil levels, as well as nonsmoking asthmatics with high blood eosinophil levels (*n* = 17). Sex, age, pack‐years, and BMI on this cohort can be found in the supplementary information datasets (Dataset‐1).

### 2.4 Blood sampling in COPD and asthma cohorts

Blood samples were collected between 2003 and 2005 (Hokkaido COPD cohort study) and 2010–2012 (Hi‐CARAT). Blood was drawn in the morning after a fast of ≥12 h. Plasma was obtained by centrifugation of EDTA whole blood at 3000 rpm, 10 min and stored at −80° C. In the COPD cohort, the plasma samples were collected from different hospitals in a same manner, and all samples were stored at the Hokkaido University Hospital. In asthma cohort (Hi‐CARAT), all plasma samples were collected and stored at the Hokkaido University Hospital. Then, all samples were thawed once, aliquoted, and stored at −80° C until assayed. The median storage time for the COPD samples was 4602 days (range 4123–4804) and for asthma it was 1962 days (range 1429–2316).

### 2.5 Statistical analyses of clinical data

Differences among the groups were analyzed using Student's *t*‐test, one‐way analysis of variance, the Mann–Whitney *U*‐test, the Kruskal–Wallis test, or Fisher's exact test. Annual change in clinical parameters were estimated using linear mixed‐effects models. Exacerbation‐free survival was analyzed using the Kaplan–Meier method with the long‐rank test. Statistical significance was defined as *p* < 0.05.

### 2.6 Proteomic analysis overview

The proteome was assessed by SomaLogic (Boulder, Colorado, USA) using SOMAscan® assay v3.2.^13^, ^14^ 100 μl of samples were provided to SomaLogic for the analysis although each analysis took only a few μl. SomaLogic data analysis workflow included hybridization normalization, median signal normalization, and signal calibration to control for inter‐plate differences. Here, 77 SOMAmer probe‐sets failed quality control, leaving 1233 that represented 1238 proteins, as 35 probe‐sets could not distinguish between protein isoforms, and a further 11 probe‐sets recognized a complex of two different proteins. These are shown in the supplementary information datasets (Dataset‐9). They cover a diverse range of protein and biological functions and as such do not impact the overall pathway and functional analysis. Data were analyzed based on probe‐set abundance as expressed by SomaLogic in relative fluorescent units.

Data and statistical analysis of the SomaLogic probe‐sets (hereafter "proteins") used R (v3.4.4) functions and python (v2.7.12). Specific analyses are detailed in the supplementary information. Protein set enrichment analyses were based on gene set enrichment methodology using bespoke python scripts for calculating normalized directional enrichment scores^24^ and non‐directional scores (earth mover's distance).^25^ Exosomal marker proteins in the SOMAscan® array were identified from the ExoCarta database.^26^ Putative cell source of proteins was assessed from mRNA expression patterns in 79 human tissues using GeneAtlas U133A, gcrma data from BioGPS.^27^ All proteomic data can be found in the supplementary information datasets (Dataset‐2 to ‐8).

## 3 RESULTS

### 3.1 Patient cohorts

Patient characteristics of the COPD and asthma cohorts are summarized in Table 1. In general, COPD patients were slightly older, had a lower body mass index (BMI) and a higher smoking index, whereas the prevalence of current smokers was comparable between COPD and asthma cohorts. The differences in clinical features reflect the definition of the two diseases. However, there was overlap in both the demographic and clinical characteristics due to our subgroup selection strategy. This was by design, to include a range of blood eosinophil levels, degree of airflow limitation, and smoking index. Furthermore, we matched for age and BMI among predefined clinical subgroups within each disease (Tables S1 and S2). By taking this approach we challenged the methodology to identify systemic differences between the disease subgroups as well as to minimize effects of some potential covariances.

**TABLE 1:** Characteristics of the COPD and asthma groups

	COPD	Asthma	*p*‐value
Number of subjects	34	51	
Female sex, *N* (%)	2 (6)	21 (43)	<0.001^b^
Age (year)	67.2 ± 7.1	61.2 ± 11.6	0.009^c^
BMI (kg/m^2^)	22.9 ± 3.4	25.1 ± 5.7	0.04^c^
Smoking index at entry, pack‐years	64.9 ± 27.1	26.5 ± 27.2	<0.001^c^
Current smokers, *N* (%)	8 (24)	7 (20)	0.26^b^
FEV_1_, L^a^	1.74 ± 0.50	2.22 ± 0.71	0.001^c^
FEV_1_, % predicted^a^	62.9 ± 16.2	86.0 ± 15.2	<0.001^c^
FEV_1_/FVC, %^a^	51.1 ± 11.6	63.7 ± 12.1	<0.001^c^
DLco, % predicted	78.0 ± 21.1	102.1 ± 20.1	<0.001^c^
Kco, % predicted	66.1 ± 20.7	104.8 ± 25.6	<0.001^c^
Blood neutrophil count, cells/mm^3^	3560 (2504–4559)	4788 (3657–6182)	<0.001^d^
Blood eosinophil count, cells/mm^3^	218 (63–388)	405 (83–659)	0.03^d^
Serum total IgE, IU/ml	91 (30–138)	253 (100–472)	<0.001^d^
Comorbidities			
Any cardiovascular disease, *N* (%)	12 (35)	NA	
Ischemic heart disease, *N* (%)	4 (12)	NA	
Diabetes, *N* (%)	1 (3)	NA	
Allergic rhinitis, *N* (%)	NA	26 (51)	
Atopic dermatitis, *N* (%)	NA	8 (16)	

*Note*: Data are shown as mean ± SD, median (interquartile range), or number (%).

Abbreviations: BMI, body mass index; DLco, carbon monoxide diffusion capacity; Kco, carbon monoxide transfer coefficient; NA, not assessed.

^a^ COPD: post‐bronchodilator (salbutamol) value; Severe asthma: maximum value of FEV_1_ among four procedures (see Section 2) and corresponding FEV_1_/FVC.

^b^ Fisher's exact test.

^c^ Student's *t* test.

^d^ Mann–Whitney *U* test.

### 3.2 The plasma proteome differs between COPD and severe asthma

**FIGURE 1:**
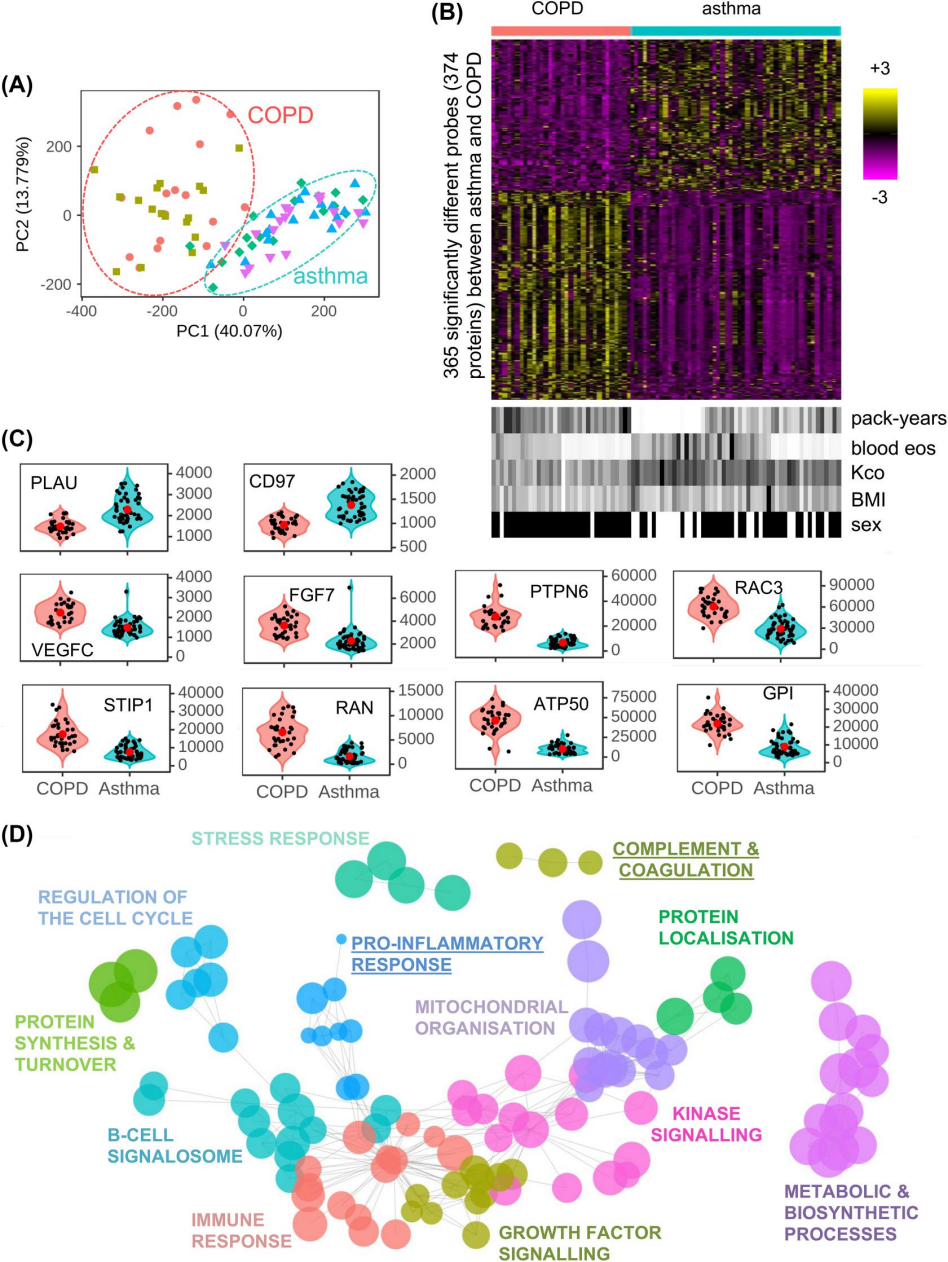
The plasma proteome differs between chronic obstructive pulmonary disease (COPD) and asthma. The plasma proteome was compared between all COPD and all asthma subjects. (A) A principal component analysis (PCA) scatterplot across all COPD and asthma subjects showing PC1 versus PC2. In the PCA scatterplots, the percent variation explained by each component is given on the axis label. COPD subjects with (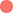) or without (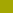) low blood eosinophils and bronchodilator reversibility. Asthma subjects with a smoking history and with high (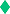) or low (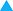) blood eosinophils, and asthma with no smoking history and high blood eosinophils (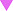). (B, upper) Heatmap of abundances for 365 significantly (*p* ≤ 0.05) different SOMAscan® probes between COPD (red bar) and asthma (blue bar) subjects. The color intensity represents row scaled (z‐score) protein abundance, with magenta as low and yellow as high abundance. The heatmap has been hierarchically clustered by row. (B, lower) For each subject, selected clinical features are given in a paired heatmap showing: smoking index (pack‐years), blood eosinophil counts (bld eos), Kco, % predicted (Kco), body mass index (BMI), and sex. Values for each feature are represented as a grayscale, with white as low and black as high except sex where females are white, and males are black. (C) Violin plots of protein abundances for selected proteins of COPD (red) and asthma (blue) subjects. Individual subjects are shown as black dots, and disease means as larger red dots. Protein abundance is given on the *y*‐axis. (D) Results from protein‐set enrichment analysis for COPD versus asthma. The results are summarized as a network, where each enriched protein‐set (*p* ≤ 0.001) is given as a node (circles) and protein‐sets with >50% of genes in common are connected by edges (lines). Representative names and arbitrary colors are given for each cluster. Node size represents the size of the difference between COPD and asthma by observed/random earth mover's distance score. Those pathways underlined are elevated in asthma as compared to COPD

To determine whether the plasma proteome could differentiate between COPD and asthma, a principal component analysis (PCA) was undertaken. This PCA scattergraph showed a clear separation between the two diseases (Figure 1A) despite using predefined groups that overlapped in their clinical and demographic features. 365 proteins were found to have different abundances between COPD and asthma (*p* ≤ 0.05 BH‐adjusted). The overall distribution of these proteins is visualized in the heatmap (Figure 1B), while the violin plots of some selected proteins show proteins that have greater abundance in COPD or asthma (Figure 1C). On the other hand, PCA scattergraph and heatmap showed no obvious differences in proteome pattern among different predefined clinical subgroups of each disease (Figure S2).

Loss of integrity of the samples over time in storage, which was several years longer for the COPD than the asthma samples, did not appear to contribute to the differences between the two diseases. Comparison of the SOMAscan® protein abundancies to single protein assessments (ELISAs and radioimmunoassay), performed shortly after sample acquisition, showed a significant Spearman's correlation (*p* ≤ 0.01) for 10 out of 11 assessments (IgE, leptin, CRP, CCL18, adiponectin, YKL40, CD5L, periostin, SPP1, but not albumin) (Figure S3). This was true when the correlation contained both asthma and COPD samples which supports the observation that the longer storage time between the two did not appear to impact the SOMAscan assay results. Furthermore, we observed only 17 and 25 proteins correlated with storage time respectively for asthma and COPD, none of which overlapped (Figure S4E and H). Finally, the individual PCAs of asthma and COPD showed no global effect of storage time (Figure S5G and H).

Additionally, while demographic characteristics, such as BMI and sex, varied between these diseases, no pattern with protein abundance of the 365 differentially expressed proteins was observed when they were aligned to the heatmap (Figure 1B). Indeed only 3, 5, and 7 proteins differ between the two cohorts for age, BMI and sex, respectively. This did not represent an enrichment over background (hypergeometric test, *p* < 0.05). Finally, to confirm that the asthma versus COPD differences were not driven by potential confounders, we plotted the five most highly significant asthma versus COPD proteins against age and gender (Figure S6A and B). These showed these confounders did not correlate within disease, for example, the youngest asthma patient had comparable protein abundance to the oldest, and this was also true for COPD. Finally, simultaneous correction for these three confounders did not alter substantially the results: there were 365 significant proteins when uncorrected, and 383 when corrected, with 349 in common (data not shown).

We also evaluated these covariances further using the whole proteome, also including assessment of inhaled corticosteroid (ICS) dose, or oral corticosteroid (OCS) dose (asthma only). Only a few proteins correlated with these covariances, including blood eosinophils (Figure S4). Furthermore, we found that in all cases they did not drive any clustering of the subjects, as visualized by overlaying values of the co‐variants onto a PCA of the proteome (Figure S5). Smoking history (pack‐years), a known risk factor for COPD, was also explored as a potential confounder. No protein was found to correlate with pack‐years either in asthma or COPD (Figure S4D) nor drive the PCA of the proteome (Figure S5I and J). A plot of the five most highly significant asthma versus COPD proteins against pack‐years clearly showed the separation between the diseases but no pattern with pack‐years (Figure S6C). Comorbidities were unlikely to drive the differences as very few individuals had co‐existing cardiovascular or ischemic heart disease or diabetes. These analyses suggest that any difference observed between or within the diseases should be driven mainly by the disease pathology.

We identified 143 networks of enriched/altered protein‐sets between COPD and asthma (*p* ≤ 0.001). These were grouped and labeled based on the main pathway observed in the network (Figure 1D). Networks that had higher protein abundances in asthma included complement & coagulation (representative protein PLAU) and pro‐inflammatory response (CD97). While those higher in COPD included growth factor signaling (VEGFC, FGF7); stress response (STIP1); regulation of cell cycle (RAN, PTPN6); mitochondrial organization (ATP50); kinase signaling (RAC3); and protein localization (GPI) (Figure 1C and D). These networks were reflected in the Ingenuity Pathway Analysis (IPA) functions analysis (Figure S7A). While IPA upstream modulators analysis found the main drivers of the response for COPD were IFNγ and TGFβ1, and involvement of the oxidative stress transcriptional regulators (BCL2L1, NFE2L2/Nrf2), and extracellular matrix regulators (BMP6, ITGB6) (Figure S7B), main drivers of response for asthma were pro‐inflammatory mediators (TNF, IL‐1β, IL‐6) and the transcriptional regulators (HDAC, CAV1, MAP4k4, Wnt, and SOX2).

### 3.3 The plasma proteome defines four distinct endotypes within severe asthma

**FIGURE 2:**
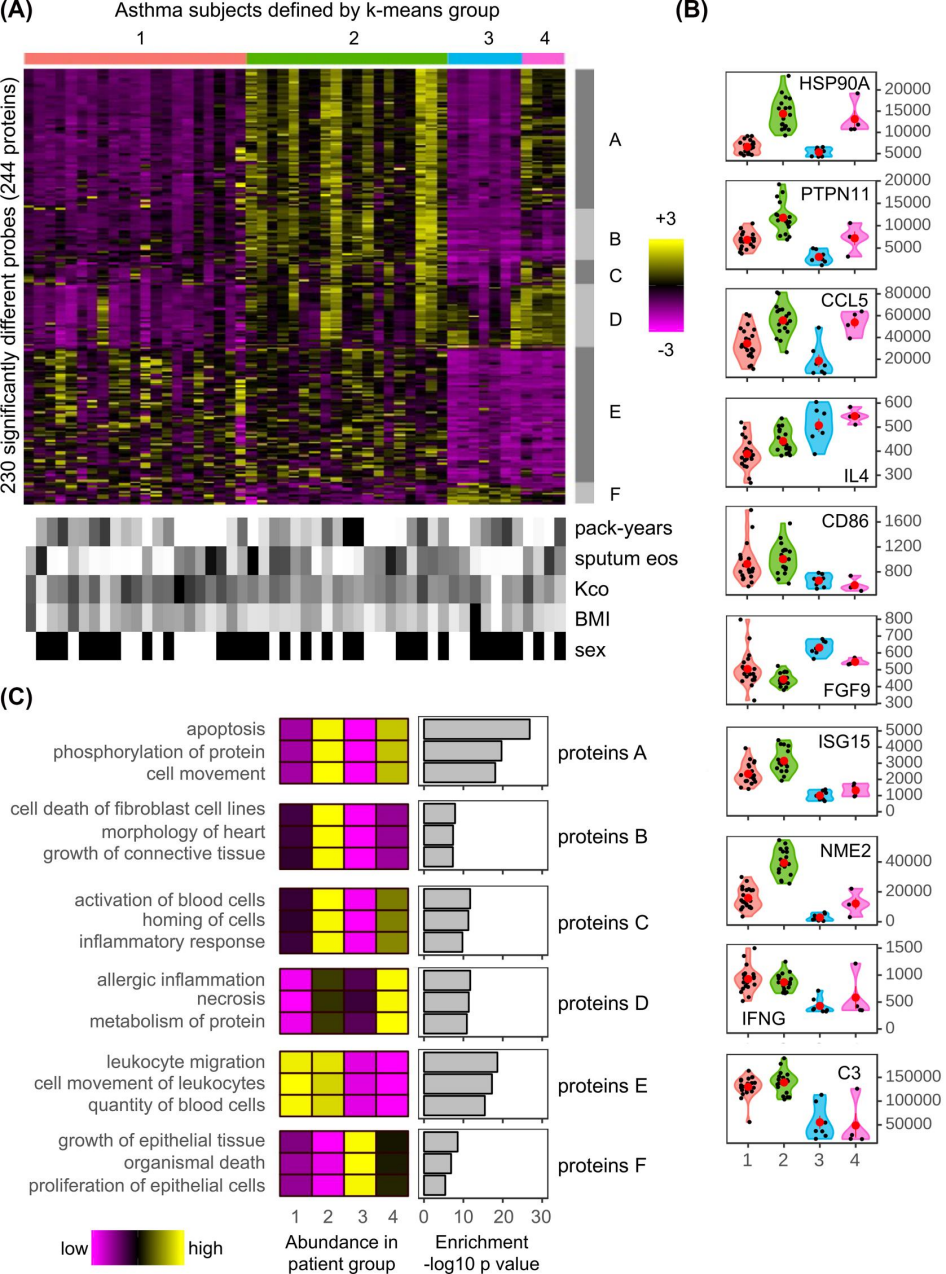
The plasma proteome defines four distinct endotypes within severe asthma subjects. (A, upper) Heatmap of the abundances for the 230 significantly (*p* ≤ 0.05) different SOMAscan® probes between any combination of the four, asthma *k*‐means subject groups. Here and in all panels, Asthma‐1 to ‐4 are colored red, green, blue, and pink, respectively. The color intensity represents row scaled (z‐score) protein abundance, with magenta as low and yellow as high abundance. The heatmap has been hierarchically clustered by row and column. Six protein groups (Proteins‐A to ‐F) with different profiles have been highlighted with gray side bars. (A, lower) For each subject, selected clinical features are given in a paired heatmap. Showing: smoking index (pack‐years), sputum eosinophil counts (sputum eos), Kco, % predicted (Kco), body mass index (BMI), and sex. Values for each feature are represented as a grayscale, with white as low and black as high except for sex where white denotes females and black males. (B) Violin plots of protein abundances for selected probes of the four asthma clusters. Individual subjects are shown as black dots, and group means as larger red dots. Protein abundance is given on the *y*‐axis. (C) Ingenuity Pathway Analysis (IPA) diseases and functions analysis for each of the six protein groups (Proteins‐A to ‐F). The function of lowest *p*‐value, for each of the top three categories is shown. The heatmaps (left) show the median abundance for all probes within each category. The bar charts (right) show the associated IPA *p*‐value (–log10) for each category

The heatmap of the COPD‐asthma differentially expressed proteins showed some clustering with smoking index and blood eosinophil levels. This suggested that there may be within‐disease endotypes. This was investigated using *k*‐means clustering of the whole proteome. Four distinct groups were observed by PCA analysis in asthma (Asthma‐1 to ‐4) (Figure S8A). These were defined by 230 proteins. These split into six blocks (Proteins‐A to ‐F) that differed between any combinations within the asthma groups (*p* ≤ 0.05 BH‐adjusted). The distributions of the proteins are shown in the heatmap (Figure 2A, for full list see Dataset‐8) and examples in violin plots (Figure 2B). The individual subject's characteristics indicates smoking index, sputum eosinophil levels, baseline diffusion capacity (carbon monoxide transfer coefficient [Kco]), BMI, and sex did not drive the clustering. The overall clinical and demographic characteristics of these subgroups are shown (Table 2). Functions and specific upstream modulator analysis are shown (Figures 2C and S8C, respectively). Together these show the characteristics of the four endotypes.

**TABLE 2:** Clinical characteristic of the four asthma *k*‐means clusters

	Asthma‐1	Asthma‐2	Asthma‐3	Asthma‐4	*p*‐value
Number of subjects	21	19	7	4	
Female sex, *N* (%)	9 (43)	8 (42)	2 (29)	2 (50)	0.89^b^
Age (years)	65.1 ± 9.2	58.7 ± 14.1	60.7 ± 11.3	53.3 ± 4.7	0.05^c^
Age at asthma onset (year)	47.0 ± 15.3	40.5 ± 14.3	39.3 ± 16.1	35.8 ± 16.3	0.09^c^
Smoking index, pack‐years	24.5 ± 24.2	29.0 ± 30.7	31.6 ± 27.3	16.6 ± 31.6	1.00^c^
BMI (kg/m^2^)	26.5 ± 5.2	23.3 ± 3.1	26.5 ± 11.0	23.7 ± 5.0	0.41^c^
Aspirin sensitivity, *N* (%)	4 (19)	4 (21)	0 (0)	1 (25)	0.61^b^
Daily ICS dose (μg)	1542 ± 412	1463 ± 146	1743 ± 600	1525 ± 50	0.60^c^
Maintenance OCS use, *N* (%)	10 (48)	8 (42)	1 (14)	4 (100)	0.16^b^
AQLQ score	5.4 ± 0.9	5.5 ± 0.8	6.0 ± 1.0	4.0 ± 1.3	0.35^c^
Blood neutrophil count, cells/mm^3^	4860 (3744–6574)	4533 (3489–5812)	4788 (3944–5558)	7143 (4371–10,089)	0.53^d^
Blood eosinophil count, cells/mm^3^	405 (57–763)	435 (113–740)	129 (83–433)	450 (347–548)	0.82^d^
Serum total IgE, IU/ml	177 (96–522)	295 (157–394)	179 (90–645)	409 (279–482)	0.93^d^
Atopy, *N* (%)	10 (48)	8 (42)	2 (29)	1 (25)	0.74^b^
Sputum eosinophils, %	12.2 (0.8–25.9)	31.1 (9.4–43.4)	0.8 (0.4–14.0)	29.4 (23.4–37.1)	0.08^d^
FeNO, ppb	28 (21–58)	35 (18–55)	19 (19–30)	71 (57–112)	0.12^d^
FEV_1_, L^a^	2.06 ± 0.66	2.36 ± 0.74	2.35 ± 0.66	2.21 ± 1.00	0.38^c^
FEV_1_, % predicted^a^	84.4 ± 13.8	88.3 ± 14.1	86.4 ± 12.5	82.7 ± 31.6	0.92^c^
FEV_1_/FVC, %^a^	63.3 ± 11.1	64.8 ± 14.4	63.8 ± 7.9	60.2 ± 14.2	0.80^c^
DLco, % predicted	105.5 ± 19.4	103.4 ± 21.2	93.6 ± 15.2	93.3 ± 26.1	0.13^c^
Kco, % predicted	115.0 ± 29.5	99.7 ± 15.3	92.46 ± 27.3	97.5 ± 30.9	0.03^c^
%Low attenuation volume on chest CT	0.49 (0.14–2.95)	0.58 (0.20–3.46)	0.78 (0.33–1.63)	0.52 (0.42–6.07)	0.82^d^
Allergic rhinitis, *N* (%)	7 (33)	14 (74)	3 (43)	2 (50)	0.07^b^
Atopic dermatitis, *N* (%)	3 (14)	4 (21)	1 (14)	0 (0)	0.94^b^

*Note*: Data are shown as mean ± SD, median (interquartile range), or number (%).

Abbreviations: AQLQ, the Asthma Quality of Life Questionnaire; BMI, body mass index; DLco, carbon monoxide diffusion capacity; FeNO, fractional exhaled nitric oxide; ICS, inhaled corticosteroid; Kco, carbon monoxide transfer coefficient; OCS, oral corticosteroid;

^a^ Maximum value of FEV_1_ among four procedures (see Section 2). FEV_1_/FVC was applied as the value corresponding to the maximum FEV_1_.

^b^ Fisher's exact test.

^c^ One‐way analysis of variance.

^d^ Kruskal–Wallis test.

The largest endotype, Asthma‐1 (*n* = 21) had subjects who were older, had a later age of asthma onset, and had increased diffusion capacity than the other clusters. This group had low protein abundancies except for Proteins‐E, which reflected an inflammatory function with upstream modulators such as LPS, IL‐1β, TNF, IL‐27RA, and IFNγ (Figure S8C).

Asthma‐2 (*n* = 19) combined a pro‐inflammatory signature, with a Th2 and innate immune response with upstream modulators of IL‐1β, TNF, IL‐13, IL‐15, and IFNγ. There were high protein abundancies of Proteins‐A reflecting a stress response (such as HSP90AA1) resulting in cell apoptosis with upstream modulators APP and MAPT; Proteins‐B, related to connective tissue remodeling; Proteins‐C and ‐E describing activation and homing of immune cells indicated an inflammatory response with LPS. These subjects' exacerbation‐free rate appeared poorer, although not significant, than that for Asthma‐1 (*p* = 0.13) (Figure S8B).

Asthma‐3 (*n* = 7) was characterized by low blood and sputum eosinophils, and low FeNO levels. This group had high Asthma Quality of Life Questionnaire (AQLQ) scores. It had low abundancies of all proteins except Proteins‐F (growth and proliferation of epithelial cells) and more variable amounts of Proteins‐D (allergic inflammation). However, what was striking was the lower abundancy of proteins involved in response to infection (ISG15, NME2, IFNγ, and C3) (Figure 2B).

The smallest subpopulation Asthma‐4 (*n* = 4) was the youngest in age and had the earliest age of asthma onset. They were defined by high levels of blood neutrophils, blood and sputum eosinophils, FeNO levels, and serum total IgE. A higher percentage of these subjects were on OCS compared to the other groups. This cohort has consistently high levels of Proteins‐D whose functions analysis described allergic inflammation or allergic pulmonary eosinophilia and Proteins‐C that describe an inflammatory response. Like Asthma‐3, they had high AQLQ score and low levels of ISG15/IFNγ and C3. They also had lower Kco %‐predicted values which might be related to Proteins‐A and ‐F that were high in the stress response and extracellular remodeling proteins.

### 3.4 The plasma proteome defines three COPD endotypes reflective of annualized decline in lung diffusion capacity (Kco)

**FIGURE 3:**
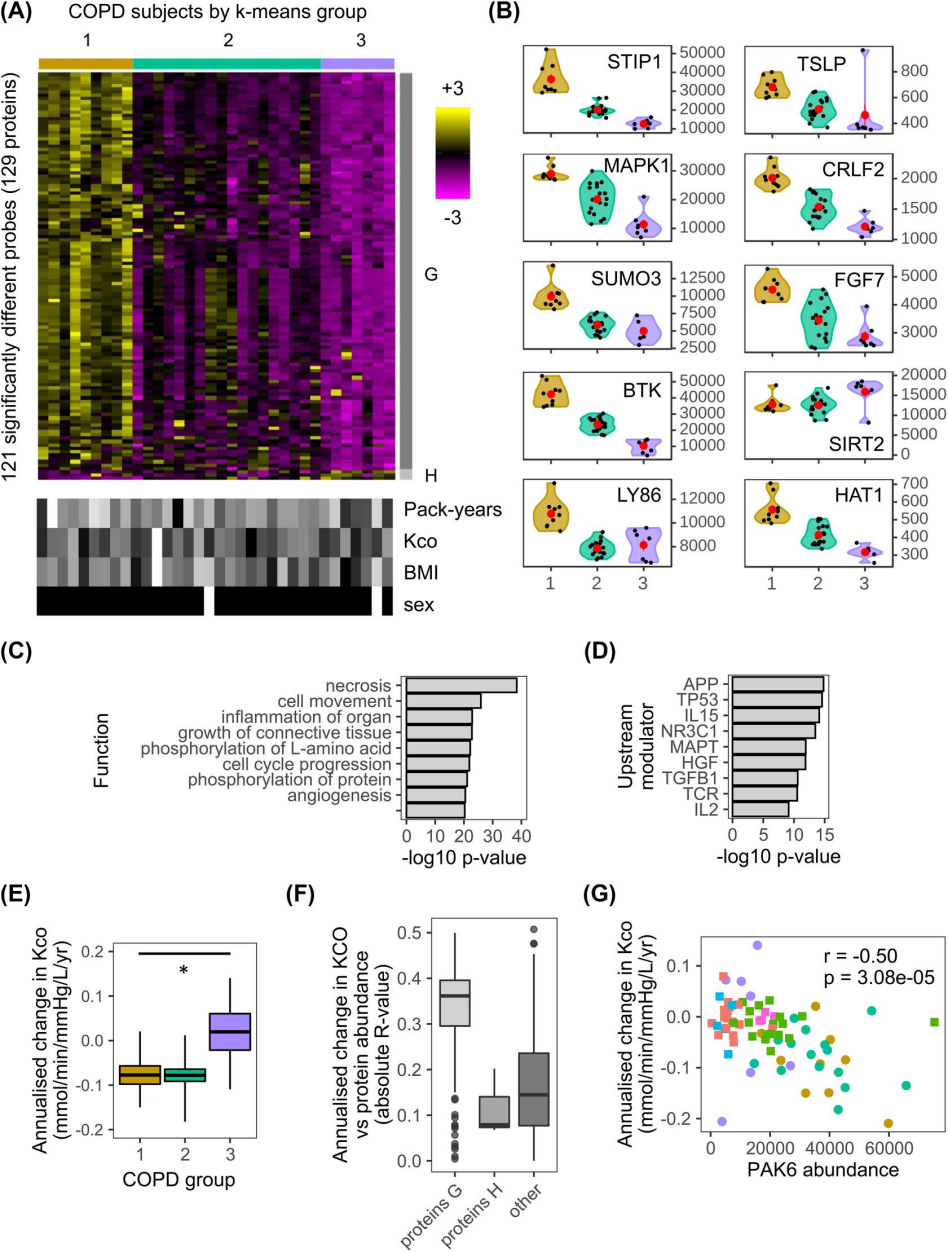
The plasma proteome defines three chronic obstructive pulmonary disease (COPD) endotypes reflective of annualized decline in lung diffusion capacity (Kco). (A, upper) Heatmap of abundances for the 121 significantly (*p* ≤ 0.05) different SOMAscan® probes between any combination of the three COPD k‐means clusters. Here and in all panels, COPD‐1 to ‐3 are colored olive green, teal and purple respectively. The color intensity represents row scaled (z‐score) protein abundances, with magenta as low and yellow as high abundance. The heatmap has been hierarchically clustered by row and column. Proteins‐G and Proteins‐H with different profiles have been highlighted with gray side bars. (A, lower) For each subject, selected clinical features are given in a paired heatmap showing: smoking index (pack‐years), Kco, % predicted (Kco), body mass index (BMI), and sex. Values for each feature are represented as a grayscale, with white as low and black as high except for sex where white denotes females and black males. (B) Violin plots of protein abundances for selected probes showing COPD‐1 to ‐3 on the *x*‐axis and protein abundance on the *y*‐axis. Individual samples are shown as black dots, and group means as larger red dots. Note HSP90A refers to the SOMAscan probe HSP90AA1. (C) Ingenuity Pathway Analysis (IPA) diseases and functions analysis for Proteins‐G. The function of lowest *p*‐value, for each of the top nine categories is shown. The bars show the associated IPA *p*‐value (−log10) for each function. (D) IPA upstream modulator analysis for Proteins‐H. The upstream modulator of lowest *p*‐value, for each of the top nine categories is shown. The bars show the associated IPA *p*‐value (−log10) for each modulator. (E) Box and whisker plots (mean and standard error) of annualized change in Kco, % predicted for COPD‐1 to ‐3 (*p < 0.05). (F) Distributions of correlations between protein abundance and annualized decline Kco, % predicted in COPD Proteins‐G and H and all other proteins (other). Correlations are given as linear regression absolute R‐values. (G) Scatterplot showing the correlation between annualized change in Kco, % predicted and PAK6 protein abundance, for Asthma‐1 to ‐4 (red, green, blue, and pink squares, respectively) and COPD‐1 to ‐3 (olive green, teal, and purple circles, respectively). The *R* value for the correlation and its associated *p*‐value is given

In contrast to the asthma picture, COPD appeared less complex. *K*‐means clustering using the whole proteome identified three groups (COPD‐1 to ‐3) with limited overlap in the PCA analysis (Figure S9A). 121 proteins drove this clustering and these defined two protein groups, Proteins‐G (*n* = 118) and ‐H (*n* = 3). The distributions of the probe‐set abundancies in the various groups are shown in the heatmap (Figure 3A) and for individual proteins in the violin plots (Figure 3B). Neither smoking index, Kco, BMI, nor sex drove the clustering. This was confirmed as baseline demographic and clinical characteristics of these COPD groups were similar (Table 3).

**TABLE 3:** Clinical characteristic of the three COPD *k*‐means clusters

	COPD‐1	COPD‐2	COPD‐3	*p*‐value
Number of subjects	10	19	5	
Female sex, *N* (%)	0 (0)	1 (5)	1 (20)	0.30^a^
Age (years)	65.7 ± 6.8	68.8 ± 6.1	63.8 ± 10.4	0.94^b^
BMI (kg/m^2^)	21.6 ± 3.1	23.3 ± 3.0	23.7 ± 5.0	0.18^b^
Smoking index at entry, pack‐years	56.3 ± 28.4	66.7 ± 22.8	75.1 ± 39.5	0.19^b^
Post‐BD FEV_1_, *L*	1.63 ± 0.54	1.85 ± 0.51	1.59 ± 0.37	0.84^b^
Post‐BD FEV_1_, % predicted	57.6 ± 20.1	66.3 ± 14.3	60.7 ± 14.6	0.50^b^
Post‐BD FEV_1_/FVC, %	0.46 ± 0.12	0.54 ± 0.10	0.51 ± 0.14	0.27^b^
Reversibility of FEV_1_, %	15.7 ± 14.0	14.7 ± 17.2	25.7 ± 17.6	0.39^b^
Reversibility of FEV_1_, ml	196.0 ± 158.8	179.7 ± 166.47	298.7 ± 152.0	0.39^b^
DLco, % predicted	74.3 ± 29.6	80.1 ± 13.6	77.9 ± 28.6	0.65^b^
Kco, % predicted	60.6 ± 26.9	68.5 ± 16.8	67.7 ± 23.0	0.43^b^
SGRQ total score	30.9 ± 15.3	27.3 ± 19.1	33.0 ± 19.1	0.97^b^
Blood neutrophil count, cells/mm^3^	3796 (3097–4886)	3392 (2189–4424)	3544 (2972–4687)	0.42^c^
Blood eosinophil count, cells/mm^3^	218 (62–344)	86 (60–386)	390 (360–400)	0.29^c^
Serum total IgE, IU/ml	82 (39–96)	97 (27–134)	327 (217–365)	0.04^c^
CT emphysema score	2.08 (0.79–2.58)	1.00 (0.42–1.29)	1.17 (0.83–1.50)	0.25^c^
Any cardiovascular disease, *N* (%)	2 (20)	8 (42)	2 (40)	0.54^a^
Ischemic heart disease, *N* (%)	1 (10)	2 (11)	1 (20)	0.79^a^
Diabetes, *N* (%)	0 (0)	1 (5)	0 (0)	1.00^a^
0–5 years longitudinal variables				
ICS Use, *N* (%)	3 (30)	1 (5)	2 (40)	0.09^a^
Exacerbation frequency, events/year	0.10 (0.00‐0.35)	0.00 (0.00–0.20)	0.40 (0.20‐0.40)	0.38^c^
Annual post‐BD FEV_1_ change, ml/year	−30.8 ± 36.9	−44.6 ± 28.9	−11.8 ± 23.4	0.56^b^
Annual DLco change, mmol/min/mmHg/year	−0.38 ± 0.34	−0.44 ± 0.26	0.02 ± 0.52	0.12^b^,*
Annual Kco change, mmol/min/mmHg/L/year	−0.08 ± 0.07	−0.08 ± 0.05	−0.005 ± 0.15	0.14^b^,**

*Note*: Data are shown as mean ± SD, median (interquartile range), or number (%).

Abbreviations: BMI, body mass index; DLco, carbon monoxide diffusion capacity; Kco, carbon monoxide transfer coefficient; Post‐BD, post‐bronchodilator; SGRQ, St. George's Respiratory Questionnaire.

*p = 0.007 for comparison between COPD‐1+2 versus COPD‐3.

**p = 0.04 for comparison between COPD‐1+2 versus COPD‐3.

^a^ Fisher's exact test.

^b^ one‐way analysis of variance.

^c^ Kruskal–Wallis test.

Proteins‐G abundancies decreased from COPD‐1 to ‐3, while Proteins‐H mainly increased. IPA functions analysis indicated Proteins‐G involvement in necrosis, cell movement, organ inflammation, growth and proliferation of connective tissue (Figure 3C) which was reflected in the upstream modulator analysis (Figure 3D). In addition, there was indication of innate/adaptive immunity drivers (IL‐15, TCR, IL‐2). 44 proteins were found to be common between Proteins‐H (36.9%, 44/119 total proteins) and Proteins‐A (59.5%, 44/74 total proteins) (Dataset‐8). COPD‐1 had the most annualized decline in Kco whereas COPD‐3 the least (Figure 3E, Table 3). Distributions of linear regression‐values of Proteins‐G showed a stronger association with annualized decline in Kco than for either Proteins‐H or all other SOMAmer proteins (Figure 3F). Similar observations were observed for baseline Kco, % predicted (Figure S9B). PAK6 had the strongest association with annualized decline in Kco (*r* = −0.50, *p* = 3.08e−05) (Figure 3G), and GDI2 had the strongest association with baseline Kco, % predicted (*r* = −0.59, *p* = 9.69e−08) (Figure S9C).

### 3.5 Role of immune and bronchial epithelial cells involved in the plasma proteome and their association with exosomal marker proteins

**FIGURE 4:**
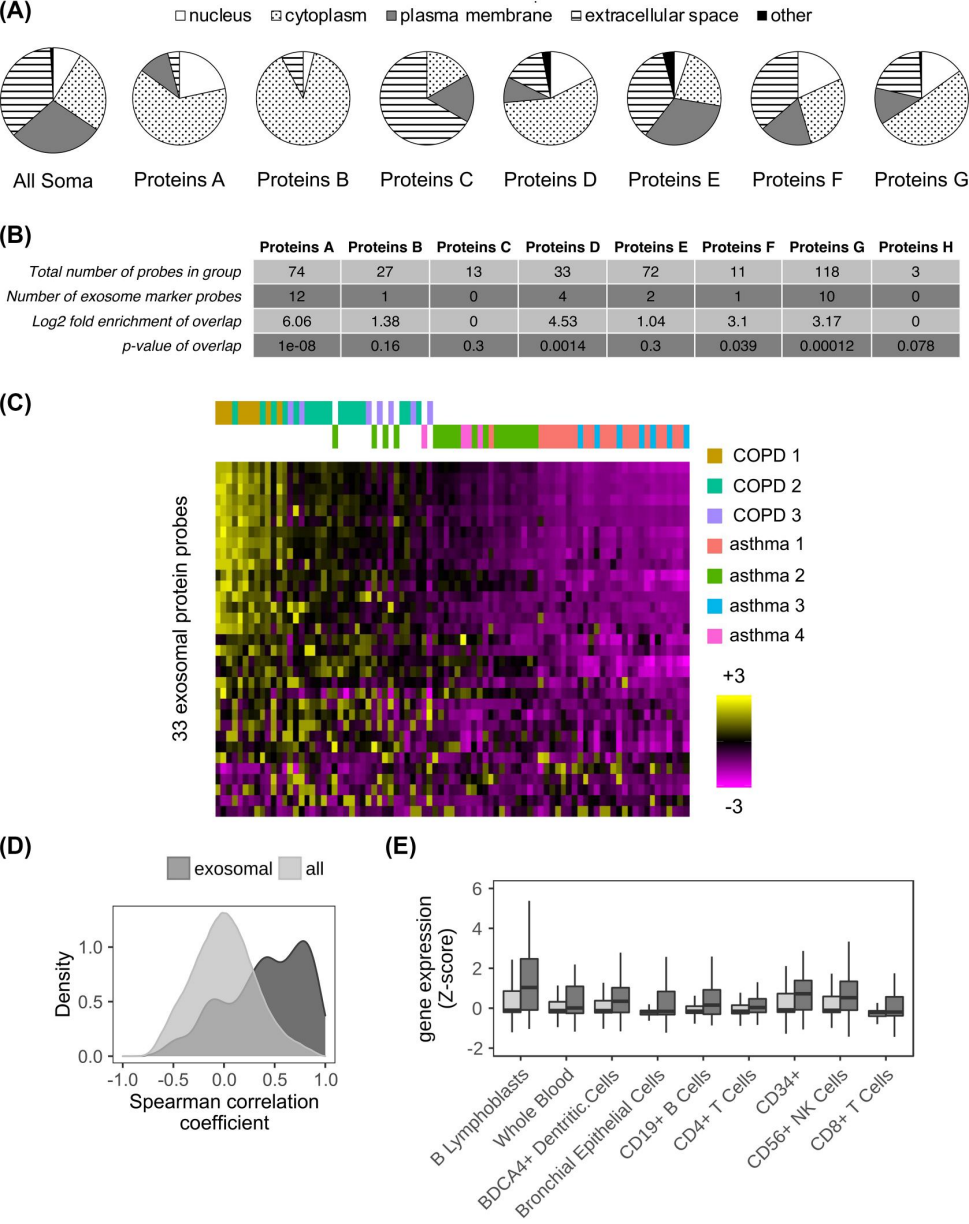
Chronic obstructive pulmonary disease (COPD) Proteins‐H and asthma Proteins‐A and ‐D all associate with exosomal marker proteins. (A) Cellular locations for all 1233 SOMAscan® probes (All Soma), COPD and asthma Proteins (Proteins‐A to ‐G) as a percentage of the group total. Each COPD and asthma Proteins group differed significantly (chi‐squared test, *p* < 0.01) from the All Soma group. The data for Proteins‐H are not shown but were composed of 1 protein from each of the following: nucleus, plasma membrane, and extracellular space. (B) Table showing the overlap between each of the COPD and asthma Proteins group, and the 33 ExoCarta exosome marker proteins. The number of overlapping proteins, log2 fold enrichment of the overlap and *p*‐value (hypergeometric test) is given. (C) Heatmap of the abundances for the 33 ExoCarta exosome marker proteins for the three COPD *k*‐means clusters (COPD 1‐3 [olive green, teal, and purple bars, respectively]) and the four asthma *k*‐means clusters (Asthma 1–4 [red, green, blue, and pink bars, respectively]). The color intensity represents row scaled (z‐score) protein abundance, with magenta as low and yellow as high abundance. The heatmap has been hierarchically clustered by row and the columns have been sorted left to right by decreasing median z‐score. (D) Density plot showing for all pairwise permutations of probes the resultant distribution of the Spearman's rank correlation coefficients. Showing separate distributions for the 33 ExoCarta exosomal marker proteins (dark gray) and all SOMAscan® probes (light gray). (E) Box and whisker plots (mean and standard deviation) for selected BioGPS tissues (*x*‐axis), showing associated gene expression values (*y*‐axis) for Proteins‐G with a significant correlation (linear regression *p* ≤ 0.001) with annualized decline Kco, % predicted (dark gray boxes) and all other SOMAscan® probes (light gray boxes). All tissues have been selected as having significantly (*p* ≤ 0.05) different expression levels between the Proteins‐G with a significant with annualized decline Kco, % predicted and non‐Proteins‐G

We wondered whether we could find out more about the cellular origin of the proteins in each Proteins group. Evaluation of the cellular localization, defined by IPA, revealed unique patterns between the groups (Figure 4A). Proteins‐C had a higher proportion of secreted proteins than all SOMAscan® proteins (67% vs. 36%). While Proteins‐A, ‐B, ‐D, and ‐G were enriched in nuclear or cytoplasmic location (85%, 92%, 74%, and 66%, respectively) compared to all SOMAmer proteins (34%). Furthermore, 80% (20 out of 25) of Proteins‐G, that had a correlation with annualized decline in Kco, were cytoplasmic/nuclear in origin. Since we are assessing plasma proteins, this suggested an active process, possibly extracellular vesicle/exosomal in nature. A significant enrichment of overlap of 33 exosomal markers was observed with COPD Proteins‐H (*p* = 1.2e−04), and asthma Proteins‐A (*p* = 1.0e−08) and ‐D (*p* = 1.4e−03) (Figure 4B). Furthermore, cluster analysis of all the subgroups indicates that an exosomal process may contribute to the endotypes observed in COPD‐1, and to a lesser extent in the COPD‐2 and ‐3 and Asthma‐2 and ‐4 (Figure 4C). These exosomal proteins have a strong correlation with each other which reflects they are secreted from similar vesicles (Figure 4D).

Evaluation of the putative cellular origin of Proteins‐G that associated with annualized decline in Kco showed a significantly higher *Z*‐score (*p* ≤ 0.05 BH‐adjusted) for adaptive (B lymphoblasts, CD19^+^ B cells) and innate (CD56^+^ NK cells and BDCA4^+^ dendritic cells) immune cells (Figure 4E). A similar pattern of immune cell origin was observed for Proteins‐G and also included bronchial epithelial cells (Figure 4E).

## 4 DISCUSSION

This study indicated that there are molecular pathways defined by systemic proteomics that differ between COPD and severe asthma even when they share clinical and demographic features such as blood eosinophilia, bronchodilator reversibility, and smoking history. This supports the concept that these diseases are fundamentally different.^28^ More differentially regulated pathways were found to be up‐regulated in COPD versus asthma subjects. These included pathways involved in metabolic and biosynthetic processes, mitochondria organization, regulation of the cell cycle, and growth factor signaling. Along with other upregulated pathways observed the data suggests that in COPD, subjects were mounting an immune driven, reparative response to stress compared to the pro‐inflammatory, complement/coagulation response seen in asthmatics. We did not assess if there were shared pathways between these diseases as we did not study the plasma proteome in a matched group of healthy controls. However, our results are consistent with a recent study that showed that plasma protein expression patterns are indicative of multiple different disease states.^29^

Like other high content ‘omics platforms, our results provide insight into the underlying biology. Here a unique pattern of abundance of Proteins‐A to ‐F defined the four asthma clusters and suggests that multiple biological processes underlie a molecular endotype. Furthermore, the results highlighted that there were both up‐ and down‐regulated pathways in the clusters. Considering the functions and upstream modulators in each Proteins group along with the proteins identified one can identify one or more key proteins that define the biology as follows:

- In Proteins‐A, the molecular chaperone heat shock proteins, such as HSP90A, is key. These proteins, sometimes called “chaperokines”, play an important role in chronic inflammation as they enable cells to survive under stress.^30^ Often overexpressed in disease, such as lung cancer, they have an anti‐apoptotic role and work to enhance disease through their involvement in the folding, activation, and assembly of key mediators of signal transduction and transcriptional regulation. These mode of actions are supported by Proteins‐A composition of a large and diverse set of enzymes involved in signaling events related to cell death and survival.
- Proteins‐B involves a type I interferon response with the induction of ISG15 (an interferon induced ubiquitin‐like protein) and NME, which interacts with HERC5 (an interferon induced E3 ligase). In humans these have been found to have a critical role in anti‐mycobacterial, but not anti‐viral immunity, by promoting IFNγ production and the post‐translational process, ISGylation, activating NK cells.^31^, ^32^, ^33^, ^34^
- Proteins‐C involved in leukocyte migration with the presence of the chemotactic cytokines CCL5 (RANTES) and CCL17 (TARC).
- Proteins‐D define allergic inflammation, with high abundance of the Th2 cytokine IL‐4 and other proteins modulated by IL‐15 (another Th2 cytokine), such as IL‐2RG (interleukin‐2 receptor gamma), an important signaling component for many cytokines including IL‐4. Additionally, some of Proteins‐D, including IL‐4 and STIP1 (stress‐induced phosphoprotein 1), reflect a response to oxidative stress through the transcription factor Nrf2 (encoded by NFE2L2 gene).^35^ Furthermore Asthma‐2 and ‐4 have high abundance and also have the highest levels of markers of Th2‐high asthma such as eosinophil counts, FeNO, and IgE.
- Proteins‐E are representative of chronic airway inflammation conferred by the strong pro‐inflammatory upstream modulator signature. Key protein markers include the chronic inflammation marker, complement C3, that has a role in innate and adaptive immunity and where human deficiency can lead to recurrent bacterial infection.^36^ Additional markers include INFγ, a Th1 response cytokine, critical for innate and adaptive immunity against viral infection.^37^
- Proteins‐F involved in the development and growth of epithelial tissue. A key protein is FGF‐9 (fibroblast growth factor 9) involved in lung development especially in regards retaining lung mesenchymal proliferation.^38^

Combining these data along with the clinical characteristics of the cluster suggests potential biological processes to target. For instance, Asthma‐2 had a poorer exacerbation‐free rate compared to Asthma‐1 and had much higher HSP90 levels which can enhance chronic inflammation. Asthma‐3 had high AQLQ score and lacked control of bacterial infection by low levels of ISG15, IFNγ, and C3. In addition to targeting Th2 cytokines in Asthma‐2 and ‐4 might be to also target the Nrf2 pathway.

Contrary to what we observed for asthma, the three COPD endotypes were defined by decreasing abundances of one large group of proteins, consistent with COPD characterized by continuous disease traits co‐existing in varying degrees, rather than by mutually exclusive subtypes.^39^ The pathway and upstream modulators analysis showed the predominant feature was cell death/apoptosis. The considerable overlap between Proteins‐G and Proteins‐A and the high levels of signaling proteins (e.g. MAPK1 and SUMO3) suggest similar importance of proteins including HSP90A. These observations align with the increase in apoptotic alveolar epithelial and endothelial cells observed in the lungs of COPD patients.^40^ Furthermore, the high abundance of proteins involved in oxidative stress (e.g. STIP1) aligns with oxidative stress proposed to be involved in the development of COPD.^41^, ^42^ While these events could be initiated by cigarette smoking, we did not see any clear association with smoking history.

We found that a subset of COPD Proteins‐G positively correlated to annualized decline in Kco %‐predicted and appeared to originate from B lymphoblasts and to a lesser extent mature CD19^+^ B cells, which suggests that their origin is non‐lymphoid tissue.^43^ These results reflect increased number of B cells previously observed in bronchial biopsies and lung tissues.^44^, ^45^, ^46^ Our results support the previous suggestion of an immune response role in COPD^47^ and that B cells are strongly linked to the emphysema phenotype.^48^ Key proteins related to this are the tyrosine kinase, BTK, that has a key role in B cell development, TSLP, originally identified as key to support B cell lymphopoiesis, and its receptor CRLF2 which is expressed on B cells.^49^

The data suggest a role of innate/adaptive immunity in lung function decline possibly related to infection as one correlate, PAK6, a protein that associates with susceptibility to childhood pneumonia^50^ and reported to be an important factor in the early origins of COPD.^51^ It is therefore possible that the B cell response in this group may be related to the host response to the lung microbiome.^52^

Proteins‐H were mainly elevated in the small COPD‐3 cluster, that also had the lowest levels of Proteins‐G. Proteins‐H were biologically interesting: TNFRSF14 reported to control TSLP drives pulmonary fibrosis.^53^ C3 linked to the control of bacterial infection^36^; SIRT2, reported to be a candidate gene for COPD, associates with FEV_1_.^54^ While a preliminary finding in a small number of COPD subjects, this latter observation supports the utility of applying large‐scale proteomic data to genome‐wide association studies.^55^ Overall, more subjects in this cluster are needed to understand the clinical impact of this observation although they had the lowest annualized Kco, % predicted of the three groups.

We observed that many plasma proteome proteins were cytoplasmic or nuclear in nature. Some of these proteins could have been released due to apoptotic or necroptotic death as these events were identified in our functions, pathways, and upstream modulator analyses. However, we observed only a few of the protein types reported to be released from myeloid cells during cell death in vitro.^56^ Further work is required to define proteins that may be released from non‐myeloid cells undergoing apoptotic or necroptotic death and to understand if any of the plasma proteome is derived from them.

We did find an association of some of the plasma Proteins groups and indeed unexpectedly nuclear proteins, with exosomal marker proteins, suggesting the importance of extracellular vesicles (apoptotic bodies, microvesicles, or exosomes) in COPD or asthma. A mechanism by which nuclear proteins are loaded into exosomes has recently been proposed^57^ although it remains to be determined if such a mechanism does occur in non‐cancerous cells. There is growing support for a role of these vesicles in asthma.^58^, ^59^ Our results suggest that the exosomal proteins are representative of allergic inflammation and higher sputum eosinophils, and supports the role of exosome secretion by eosinophils in asthma pathogenesis.^60^ Elevated exosomes have been reported to be elevated in stable COPD or COPD exacerbation and correlated with plasma biomarkers of systemic inflammation.^61^ Overall, this study suggests the importance of extracellular vesicles in COPD and asthma endotypes, especially those derived from innate/adaptive immune cells.

Our results indicate that the SOMAscan abundance data compare well to other protein analysis platforms. However, use of single protein assessment measures to validate the SOMAscan data has some limitations as follows: a) not all proteins in the SOMAscan array have suitable low throughput options to validate a result; b) the SOMAscan platform has a large dynamic range which may not always be the case for other platforms; c) in general single assessment measures have larger coefficient of variability (CoV) than SOMAscan platform with a 3%–4% CoV^62^ which drives greater precision in any analysis; d) and finally subtly different epitopes may be assessed between the two methods which is common even between ELISAs to the same protein.

Although the average storage time of the samples before the SOMAscan assay differed between asthma and COPD cohorts, the data show that this does not contribute to the differences we detect. Analysis showed that within either cohort less than 2.0% of the total proteins assessed showed an apparent weak correlation with time in storage before assay. Furthermore, because the samples from the two cohorts were collected over similar lengths of time (887 and 681 days respectively for asthma and COPD), one would have expected to see the same proteins correlate with time in storage in both cohorts. However, it was different proteins in each cohort, indicating that these few correlations could be spurious.

In summary, this analysis shows the utility of large‐scale plasma proteome analysis combined with the integration of clinical, disease and bioinformatic sciences. While a pilot study in nature, this non‐invasive method that simultaneously evaluates levels of numerous proteins has potential for: a) repository of plasma biomarkers for discovery; b) the definition of molecular endotypes; c) providing new insights into the complex biology of multiple molecular pathways and the identification of potential therapeutic protein targets; d) the role of different cells and or the cell processes that characterize the molecular endotypes; and finally e) linking genetic traits and protein expression. The potential that molecular understanding identified by proteins, rather than mRNA‐driven, provides a basis for addressing new ways to target the right pathobiology in the right patient cohort.

## CONFLICT OF INTERESTS

Rose A. Maciewicz was an employee of AstraZeneca and owns stock in this company. Masaru Suzuki has received lecture fees from AstraZeneca, Boehringer Ingelheim, Novartis, and GlaxoSmithKline. Satoshi Konno has received grants from AstraZeneca, Boehringer Ingelheim, KYORIN Pharmaceutical, Novartis, and Japan Allergy Foundation during the conduct of the study, and has received lecture fees from AstraZeneca and Boehringer Ingelheim. Masaharu Nishimura has received grants from AstraZeneca, Boehringer Ingelheim, KYORIN Pharmaceutical, and MSD during the conduct of the study. John J. Cole, Hironi Makita, and Hiroki Kimura have no relevant conflicts of interest.

## Supporting information

Supporting Information S1

Supporting Information S2

## Data Availability

The data that support the findings of this study are available from the corresponding author upon reasonable request.
