# Supplementary material for: Large‐scale plasma proteomics can reveal distinct endotypes in chronic obstructive pulmonary disease and severe asthma
Source: Clin Transl Allergy. 2021 Dec 20;11(10):e12091. doi: 10.1002/clt2.12091 (PMC8686766; doi:10.1002/clt2.12091)

**Large-scale plasma proteomics can reveal distinct endotypes in COPD and severe asthma**

Masaru Suzuki^1^*, John J. Cole^2^*, Satoshi Konno^1^, Hironi Makita^1,3^, Hiroki Kimura^1^, Masaharu Nishimura^1,3^, Rose A. Maciewicz^2,4^

^1^ Department of Respiratory Medicine, Faculty of Medicine and Graduate School of Medicine, Hokkaido University, Sapporo, Japan

^2^ GLAZgo Discovery Centre, University of Glasgow, Glasgow, UK

^3^ Hokkaido Medical Research Institute for Respiratory Diseases, Sapporo, Japan

^4^ Respiratory, Inflammation and Autoimmunity, Innovative Medicines and Early Development Biotech Unit, AstraZeneca, Gothenburg, Sweden

*These authors contributed equally and are co-first authors.

**Supporting Information**

**Methods**

**Patients cohorts**

The protocols for the Hokkaido COPD cohort, Hi-CARAT, and this study were approved by the ethics committee of Hokkaido University School of Medicine (med02-001) and Hokkaido University Hospital (009-0025, 015-0336), respectively. They were performed in accordance with the Declaration of Helsinki. All subjects provided written, informed consent with an additional opt-out consent for this study. The study design, standardized sampling methods and data collection for the two cohorts have been described.^1-8^ Information on comorbidities was collected at baseline by a questionnaire and a medical chart review.

**Selection of predefined clinical subgroups of COPD**

Among 279 subjects participated in the Hokkaido COPD cohort study, plasma sample was available in 261 subjects. Among them, there were 17 subjects who had high blood eosinophil levels (>300/μL at baseline) and bronchodilator reversibility (ΔFEV_1_ ≥200mL and ≥12% after inhalation of 400 µg of salbutamol, the average value for three visits taken during the 1^st^ year). On the other hand, there were 47 subjects who had low eosinophil levels (<100/μL at baseline) and did not have bronchodilator reversibility. From those, 17 subjects were selected by matching age and BMI (Figure S1).

**Selection of predefined clinical subgroups of severe asthma**

Among 127 subjects participated in the Hi-CARAT study, 114 subjects had age of onset after 16 years old. Among them, there were 17 subjects who had smoking history (pack-years >10) and high blood eosinophil levels (>300/μL at baseline). Whereas there were 20 subjects who had smoking history and low blood eosinophil levels (<150/μL at baseline), and 17 subjects were selected by matching age and BMI. On the other hand, there were 29 subjects who had no smoking history (pack-years <5) and high blood eosinophil levels, and 17 subjects were selected by matching age and BMI (Figure S1).

**Measurement of proteins by single assays**

Plasma leptin and adiponectin levels (COPD samples) were quantified using Quantikine ELISA Kits (R&D Systems, Minneapolis, MN) or in asthma serum by double-antibody radioimmunoassay and latex immune nephelometry (SRL, Inc., Japan). Serum levels of CCL18, YKL-40, and osteopontin (SPP1) were quantified using Quantikine ELISA Kits (R&D Systems, Minneapolis, MN), and periostin levels by ELISA as described previously.^5^ AIM/CD5L was measured by ELISA kit (Trans Genic, Inc., Japan), and serum levels of albumin, IgE, and CRP were assessed in the Hokkaido University Hospital. Serum was collected at the same time as plasma and stored at -80$^{\circ}$C until analysis. All single assays were performed shortly after sample acquisition.

**Proteomic analysis**

Data and statistical analysis used R (v3.4.4) functions identified in bold italics. Principal Component Analysis (PCA) used ***prcomp***. For visualization purposes the PCA units are shown as 1/1000 of the relative fluorescent units. Hierarchically clustered heatmaps were generated using ***heatmap.2***, with the distance method Spearman and cluster method Average. For visualization purposes only, per protein outlier samples (*>*1.5 times the interquartile range) were set to 1.5. The data was scaled as per protein (row) z-scores using ***scale***. Paired clinical heatmaps were normalized so that values ranged from 0 to 1 per feature, using the formula (x-max)/max-min.

Differential protein-sets abundancies were identified using ***t.test*** (unpaired two tailed t-test equal variance) and log2 fold changes used group means. Benjamini-Hochberg (BH) adjustment was performed using ***p.adjust*** (p≤0.05). Spearman correlation coefficients (SCC), linear regression R values, and associated p-values were calculated using the python (v2.7.12) scipy packages spearmanr and linregress. Adjustment for covariance effects used the R package ***sva-ComBat***.

Protein-set enrichment analysis was performed on SOMAmer probe identifiers after mapping to UniProt identities^9^ and used a bespoke python (v2.7.12) script for calculating normalized enrichment score (NES)^10^ to identify protein-sets that might be skewed towards up- or down-regulation. Earth Mover Distance (EMD) was performed to identify protein-sets that were different but with no overall direction using a bespoke python script, comparable to that described.^11^ For each protein-set two matrices of protein abundancies were generated: the first matrix included abundancies for all COPD subjects across all proteins in the protein-set; and the second matrix included abundancies for all asthma subjects across the same proteins. All abundancies were pre-scaled as z-scores. The EMD score was then calculated by comparing the two matrices using the using the python (v2.7.12) package pyemd (https://github.com/wmayner/pyemd). Significance (p≤0.001) was calculated by determining on how many occasions out of 1000 random trials the observed NES or EMD score was greater than the random.

For protein-set enrichment, a combination of the following MsigDB collections^12^ were used in the analysis: canonical pathways, BIOCARTA, KEGG, REACTOME and GO. Here proteins were aligned to their gene identification. Only protein-sets with 15-250 proteins were included. Due to ambiguity, SOMAmer probe identifiers that refer to protein complexes were excluded from this analysis. To give a measure of biological or knowledge-based pathway redundancy, the results were plotted as a network, with nodes as significant protein-sets, and edges joining two protein-sets where either included >65% of the proteins of the other. For clarity, singleton protein-sets were removed. The network layout used network, under default settings. Node clusters were identified using the MCL clustering function of Biolayout Express^13^, with inflation of 1.7, pre-inflation of 1.7, scheme of 6.^11^

Ingenuity Pathways Analysis (IPA, QIAGEN Inc.)^14^ was performed on SOMAmer probe identifiers after mapping to UniProt identities.^9^ Where identifiers referred to complexes, each protein in the complex was listed. Upstream modulator, and disease and functions analysis were performed using the settings: Ingenuity Knowledge Base (genes only); direct and indirect relationships where confidence was experimentally observed or high (predicted); excluded endogenous chemicals. For the COPD versus asthma comparisons, fold changes and p-values were included to allow IPA to calculate activation states.

K-means clustering of COPD and asthma subjects used ***kmeans*** where the sum of squares was calculated for each of 1-15 k-means clusters and the optimal number of clusters identified using the elbow method (data not shown). 100 iterations of k-means clustering (using a different random seed each time) was performed, and clusters, which occurred >50% of the iterations, were selected. Protein sub-cellular locations used the IPA^14^ summary function. SOMAmer probe identifiers that refer to protein complexes were included, and each protein in the complex listed.

Exosomal marker proteins in the SOMAscan array were identified from the ExoCarta database.^15^ When determining overlapping proteins with the exosome marker proteins, SOMAmer probe identifiers of protein complexes with more than one exosome marker were counted once. Overlap p-values and fold enrichments used ***phyper***. Putative cell source of proteins was assessed from mRNA expression patterns in 79 human tissues usingGeneAtlas U133A, gcrma data from BioGPS.^16^ To compare between the tissues, the expression data was transformed into per gene z-scores using scale. Differential expression analysis used ***t.test*** (unpaired two tailed t-test, equal variance). SOMAmer probe identifiers of protein complexes were included, and each protein in the complex listed.

**References**

1. Konno S, Taniguchi N, Makita H, et al. Distinct phenotypes of smokers with fixed airflow limitation identified by cluster analysis of asthma. Ann Am Thorac Soc 2018;15(1):33-41.
2. Suzuki M, Makita H, Konno S, et al. Asthma-like features and clinical course of chronic obstructive pulmonary disease. An analysis from the Hokkaido COPD cohort study. Am J Respir Crit Care Med 2016;194(11):1358-1365.
3. Makita H, Nasuhara Y, Nagai K, et al. Characterisation of phenotypes based on severity of emphysema in chronic obstructive pulmonary disease. Thorax 2007;62(11):932-937.
4. Nishimura M, Makita H, Nagai K, et al. Annual change in pulmonary function and clinical phenotype in chronic obstructive pulmonary disease. Am J Respir Crit Care Med 2012;185(1):44-52.
5. Kimura H, Konno S, Nakamaru Y, et al. Sinus computed tomographic findings in adult smokers and nonsmokers with asthma. Analysis of clinical indices and biomarkers. Ann Am Thorac Soc 2017;14(3):332-341.
6. Kimura H, Konno S, Makita H, et al. Prospective predictors of exacerbation status in asthma over a 3-year follow-up. Clin Exp Allergy 2018;48(9):1137-1146.
7. Suzuki M, Makita H, Őstling J, et al. Lower leptin/adiponectin ratio and risk of rapid lung function decline in chronic obstructive pulmonary disease. Ann Am Thorac Soc 2014;11(10):1511-1520.
8. Goudarzi H, Konno S, Kimura H, et al. Impact of abdominal visceral adiposity on adult asthma symptoms. J Allergy Clin Immunol Pract 2019;7(4):1222-1229.
9. The UniProt Consortium. UniProt: the universal protein knowledgebase. Nucleic Acids Res 2017;45(D1):D158-D169.
10. Subramanian A, Tamayo P, Mootha VK, et al. Gene set enrichment analysis: a knowledge-based approach for interpreting genome-wide expression profiles. Proc Natl Acad Sci U S A 2005;102(43):15545-15550.
11. Nabavi S, Schmolze D, Maitituoheti M, et al. EMDomics: a robust and powerful method for the identification of genes differentially expressed between heterogeneous classes. Bioinformatics 2016;32(4):533-541.
12. [Liberzon](javascript:;) A, [Subramanian](javascript:;) A, Pinchback R, et al. Molecular signatures database (MSigDB) 3.0. Bioinformatics 2011;27(12):1739-1740.
13. Theocharidis A, van Dongen S, Enright AJ, et al. Network visualization and analysis of gene expression data using BioLayout Express(3D). Nat Protoc 2009 ;4(10):1535-1550.
14. Krämer A, Green J, Pollard J Jr, et al. Causal analysis approaches in Ingenuity Pathway Analysis. Bioinformatics 2014;30(4):523-530.
15. Keerthikumar S, Chisanga D, Ariyaratne D, et al. ExoCarta: a web-based compendium of exosomal cargo. J Mol Biol 2016;428(4):688-692.
16. Su AI, Wiltshire T, Batalov S, et al. A gene atlas of the mouse and human protein-encoding transcriptomes. Proc Natl Acad Sci U S A 2004;101(16):6062-6067.

**Table S1. Characteristics of the predefined clinical subgroups of COPD**

|  | COPD with high blood eosinophils and bronchodilator reversibility | COPD with low blood eosinophils and without bronchodilator reversibility | p-value |
| --- | --- | --- | --- |
| Number of subjects | 17 | 17 |  |
| Female sex, N (%) | 1 (6) | 1 (6) | 1.00^†^ |
| Age, years | 66.2 ± 7.3 | 68.1 ± 6.9 | 0.45^‡^ |
| BMI, kg/m^2^ | 23.6 ± 3.6 | 22.1 ± 3.0 | 0.21^‡^ |
| Smoking index at entry, pack-years | 64.5 ± 27.3 | 65.1 ± 27.7 | 0.96^‡^ |
| Post-BD FEV_1_, L | 1.64 ± 0.29 | 1.84 ± 0.65 | 0.25^‡^ |
| Post-BD FEV_1_, % predicted | 59.2 ± 11.8 | 66.7 ± 19.3 | 0.18^‡^ |
| Post-BD FEV_1_/FVC, % | 48.7 ± 10.8 | 53.6 ± 12.1 | 0.22^‡^ |
| Reversibility of FEV_1_, % | 29.1 ± 14.5 | 4.1 ± 2.9 | <0.001^‡^ |
| Reversibility of FEV_1_, mL | 338.0 ± 111.5 | 66.0 ± 53.9 | <0.001^‡^ |
| DLco, %predicted | 80.8 ± 19.0 | 75.3 ± 23.3 | 0.46^‡^ |
| Kco, %predicted | 70.1 ± 20.0 | 62.0 ± 21.2 | 0.26^‡^ |
| SGRQ total score | 27.3 ± 19.1 | 33.0 ± 19.1 | 0.08^‡^ |
| Blood neutrophil count, cells/mm^3^ | 3575 (2880-4330) | 3101 (2090-4572) | 0.52^§^ |
| Blood eosinophil count, cells/mm^3^ | 390 (360-444) | 63 (50-72) | <0.001^§^ |
| Serum total IgE, IU/mL | 85 (67-250) | 97 (25-127) | 0.24^§^ |
| CT emphysema score | 1.00 (0.50-1.17) | 1.50 (0.50-2.17) | 0.36^§^ |
| Any cardiovascular disease, N (%) | 6 (35) | 6 (35) | 1.00^†^ |
| Ischemic heart disease, N (%) | 2 (12) | 2 (12) | 1.00^†^ |
| Diabetes, N (%) | 0 (0) | 1 (6) | 1.00^†^ |
| 0y-5y longitudinal variables |  |  |  |
| ICS use, N (%) | 4 (24) | 2 (12) | 0.65^†^ |
| Exacerbation frequency, events/year | 0.20 (0.00-0.40) | 0.00 (0.00-0.20) | 0.88^§^ |
| Annual post-BD FEV_1_ change,  mL/year | -23.0 ± 33.5 | -47.1 ± 26.8 | 0.33^†^ |
| Annual DLco change,  mmol/min/mmHg/year | -0.31 ± 0.46 | -0.39 ± 0.24 | 0.56^†^ |
| Annual Kco change,  mmol/min/mmHg/L/yr | -0.07 ± 0.10 | -0.07 ± 0.05 | 0.91^†^ |

Data are shown as mean ± SD, median (interquartile range), or number (%).

BMI = body mass index; Post-BD = post-bronchodilator; DLco = carbon monoxide diffusion capacity; Kco = carbon monoxide transfer coefficient; SGRQ = St. George’s Respiratory Questionnaire.

^†^ Fisher’s exact test, ^‡^ Student’s t-test, ^§^ Mann–Whitney U test.

**Table S2. Characteristics of the predefined clinical subgroups of asthma**

|  | Smoking asthma with high eosinophils | Smoking asthma with low eosinophils | Non-smoking asthma | p-value |
| --- | --- | --- | --- | --- |
| Number of subjects | 17 | 17 | 17 |  |
| Female sex, N (%) | 4 (24) | 5 (29) | 12 (71) | 0.01^‡^ |
| Age, yr | 59.4 ± 11.0 | 65.1 ± 12.0 | 59.1 ± 11.6 | 0.24^§^ |
| Age at asthma onset, year | 41.2 ± 13.0 | 46.6 ± 16.8 | 40.1 ± 15.4 | 0.41^§^ |
| Smoking index, pack-years | 35.5 ± 19.9 | 43.8 ± 27.9 | 0.2 ± 0.6 | <0.001^§^ |
| BMI, kg/m^2^ | 25.5 ± 7.8 | 25.4 ± 3.7 | 24.4 ± 5.1 | 0.81^§^ |
| Aspirin sensitivity, N (%) | 2 (12) | 3 (18) | 4 (24) | 0.67^‡^ |
| Daily ICS dose, µg | 1471 ± 136 | 1696 ± 547 | 1450 ± 198 | 0.08^§^ |
| Maintenance OCS use, N (%) | 7 (41) | 9 (53) | 7 (41) | 0.73^‡^ |
| AQLQ score | 5.4 ± 1.0 | 5.4 ± 1.0 | 5.4 ± 1.1 | 0.99^§^ |
| Blood neutrophil count, cells/mm^3^ | 4224  (3856-5127) | 5913  (5040-7209) | 4058  (3120-6014) | 0.02^¶^ |
| Blood eosinophil count, cells/mm^3^ | 561 (405-819) | 57 (22-83) | 586 (427-825) | <0.001^¶^ |
| Serum total IgE, IU/mL | 358 (168-582) | 68 (19-195) | 310 (177-449) | 0.009^¶^ |
| Atopy, N (%) | 11 (65) | 8 (47) | 11 (65) | 0.48^¶^ |
| Sputum eosinophils, % | 24.3 (8.6-38.8) | 0.4 (0.4-1.6) | 31.6 (24.8-54.4) | <0.001^¶^ |
| FeNO, ppb | 58 (39-79) | 19 (10-22) | 49 (25-81) | <0.001^¶^ |
| FEV_1_, L^†^ | 2.41 ± 0.71 | 2.16 ± 0.71 | 2.09 ± 0.72 | 0.40^§^ |
| FEV_1_, % predicted^†^ | 83.9 ± 17.9 | 86.5 ± 14.5 | 87.5 ± 13.49 | 0.78^§^ |
| FEV_1_/FVC, %^†^ | 62.5 ± 13.48 | 61.8 ± 12.4 | 66.8 ± 10.2 | 0.43^§^ |
| DLco, % predicted | 100.7 ± 21.5 | 100.5 ± 21.5 | 105.2 ± 17.9 | 0.74^§^ |
| Kco, % predicted | 100.3 ± 22.7 | 96.6 ± 26.1 | 117.6 ± 24.2 | 0.04^§^ |
| %Low attenuation volume  on chest CT | 2.52  (0.21-4.23) | 1.01  (0.19-4.44) | 0.36  (0.22-0.54) | 0.15^¶^ |
| Allergic rhinitis, N (%) | 8 (47) | 7 (41) | 11 (65) | 0.46^‡^ |
| Atopic dermatitis, N (%) | 2 (12) | 3 (18) | 3 (18) | 1.00^‡^ |

Data are shown as mean ± SD, median (interquartile range), or number (%).

Eo = eosinophils; BMI = body mass index; ICS = inhaled corticosteroid; OCS = oral corticosteroid; AQLQ = the Asthma Quality of Life Questionnaire; FeNO = fractional exhaled nitric oxide; DLco = carbon monoxide diffusion capacity; Kco = carbon monoxide transfer coefficient.

^†^ Maximum value of FEV_1_ among four procedures (see Methods section). FEV_1_/FVC was applied as the value corresponding to the maximum FEV_1_.

^‡^ Fisher’s exact test, ^§^ one-way analysis of variance, ^¶^ Kruskal–Wallis test.

**Dataset Legends**

**Dataset-1. Patient information**

Individual demographic and clinical information for the 85 patient samples.

**Dataset-2. Protein abundances**

Complete SOMAscan dataset, showing each protein and its abundance (RFU) within each sample. Each protein is described by its Somalogic ID, UniProt ID, EntrezGeneSymbol and protein description.

**Dataset-3. Asthma groups and COPD groups comparisons**

Differential protein abundance (RFU) statistics for asthma groups and COPD groups comparisons showing RFU median for each protein in each group, and log2 fold and p-value, including Benjamini-Hochberg adjusted p-value, for comparison between groups. Comparison groups include: 1) COPD with asthma-like features versus COPD without asthma-like features; 2) asthma with high eosinophils (smoker) versus asthma with low eosinophils (smoker); 3) asthma with high eosinophils (smoker) versus asthma with high eosinophils (non-smoker).

**Dataset-4. Correlation between clinical parameters and protein abundances**.

For each protein probe on the SOMAscan array the associated t-test p-values and Benjamini-Hochberg adjusted p-values (for sex analysis only) or the Spearman’s rank correlation coefficient (SCC), p-values, and Benjamini-Hochberg adjusted p-value (age, blood eosinophils, BMI, pack-years, days in storage, ICS dose, OCS does (asthma only)) are given between protein abundance (RFU) and each of the demographic / clinical parameters.

**Dataset-5. Asthma versus COPD protein abundance comparison**

Differential protein abundance (RFU) statistics for asthma and COPD showing RFU median for each protein in asthma and COPD, and log2 fold and p-value, including Benjamini-Hochberg adjusted p-value, for comparison between asthma and COPD.

**Dataset-6. Summary of Asthma versus COPD enriched pathway clusters**

List of the significantly enriched protein ontologies and their representative cluster, and the list of protein in each cluster.

**Dataset-7. Asthma and COPD k-means patient groups**

List of the 85 patient samples and the hypothesis free group they were assigned to after k-means clustering.

**Dataset-8. Asthma and COPD k-means Proteins groups**

Assignment of SOMAscan protein probes in each Proteins group defined after k-means clustering of asthma samples or COPD samples (1 present, 0 not present).

**Dataset-9. Probe sets that failed QC or did not uniquely identify one protein**

Proteins that failed QC, where complexes, or did not separate isoforms are described by its Somalogic ID, UniProt ID, EntrezGeneSymbol and protein description.

**Supplementary Figure Legends**

**Figure S1. Flowcharts of selection of clinically predefined subgroups of COPD and severe asthma**

**Figure S2. The plasma proteome does not differentiate within COPD or within asthma subjects who have features of both diseases**

The plasma proteome was assessed in COPD subjects with and without asthma-like features and in asthma subjects with a smoking history and with high or low blood eosinophils, and asthma with no smoking history and high blood eosinophils. (**A**) Within-disease PCA analysis. COPD subjects with a smoking history with and without asthma-like features are denoted (◯ red and □ olive green$,$respectively); while asthma subjects with a smoking history and with high (♢ green) or low (△ blue) blood eosinophils, and asthma with no smoking history and high blood eosinophils (▽ pink). (**B**) Heatmap of abundances of 1,233 SOMAscan probes per individual subject compared within-disease. The color intensity represents row scaled (z-score) protein abundances, with magenta as low and yellow as high abundance. Heatmaps are hierarchically clustered by row.

**Figure S3. Consistency between SOMAscan and relative protein abundances by single assays in asthma and COPD subjects**

Relative abundances are shown for (**A**) IgE, (**B**) leptin, (**C**) CRP, (**D**) CCL18, (**E**) adiponectin (COPD), (**F**) adiponectin (asthma), (**G**) YKL40, (**H**) CD5L, (**I**) Periostin, (**J**) SPP1, and (**K**) albumin. COPD subjects with a smoking history with and without asthma-like features are denoted (◯ red and □ olive green$,$respectively); while asthma subjects with a smoking history and with high (♢ green) or low blood eosinophils (△ blue), and asthma with no smoking history and high blood eosinophils (▽ pink). (**L**) Summary table for (**A-K**) showing for each protein the number of samples, the SCC between the SOMAscan and relative abundances by single assays, and the p-value of the correlation.

**Figure S4. Minimal correlation of protein abundance and covariances**

Correlation analysis between protein abundance and various parameters. Showing correlations with (**A**) age, (**B**) BMI, (**C**) ICS dose, (**D**) smoking history (pack years), (**E**) days sample was in storage, and (**G**) blood eosinophils. For each plot the Spearman’s rank correlation coefficient (SCC) between protein abundance and clinical parameter in all COPD subjects is given on the x-axis and in all asthma subjects on the y-axis. Data (dots) is shown for each of the 1,233 valid SOMAscan probes. Highlighting proteins that correlate with clinical parameters significantly (SCC associated p-value, Benjamini-Hochberg-correction, p<0.05) in both COPD and asthma (green), COPD only (red), asthma only (blue) and neither COPD nor asthma (black). (**F**) Showing the clinical parameter sex and the t-test p-value (-log10) for protein abundance vs sex on the x- and y-axis. (**H**) Table summarizing the results of (A-G). Showing: the number of proteins that were significant in both COPD and asthma (Benjamini-Hochberg-corrected p-value, p<0.05), COPD only, asthma only and neither COPD nor asthma.

**Figure S5. Lack of any correlation between various covariances in either COPD or severe asthma subjects**

PCA across all SOMAscan proteins for COPD (left panels) and asthma (right panels). Subjects are colored by (**A, B**) age, (**C, D**) BMI, (**E, F**) sex, (**G, H**) days in storage, (**I, J**) smoking history (pack-years), (**K, L**) inhaled corticosteroid (ICS) dose and (**M**) oral corticosteroid (OCS) dose.

**Figure S6. Lack of correlation with five most highly significant Asthma versus COPD proteins against possible confounders age, sex, and pack-years**

(**A**) Scatterplot showing the relationship between patient age and protein abundance, for the 5 most highly significant asthma vs. COPD proteins (CDH1, IL1R, RAC3, PTP1C, ATPO). Asthma and COPD samples are red and blue, respectively. (**B**) Boxplot comparing protein abundance between male and female for COPD and asthma, for the top 5 proteins. For all 5 proteins the within asthma or COPD male vs. female comparison is not significant (adjusted p>0.05). (**C**) As (**A**) however showing pack-years.

**Figure S7. Disease and Functions analysis for differentially expressed proteins between COPD and asthma subjects**

Results from IPA diseases and functions analysis for the 365 significantly (p<0.05) different probes, between COPD and asthma subjects. Showing: functions that are enriched in COPD (**A, left**), functions that are predicted to be activated in COPD (**A, upper**), functions that are enriched in asthma (**B, left**), functions that are predicted to be activated in asthma (**B, lower**). In each plot the function of lowest p-value or z-score, for each of the top 5 categories is given.

**Figure S8. The four distinct asthma endotypes are composed of proteins that have unique upstream modulators**

(**A**) PCA scatterplot across all asthma subjects and the 230 significantly different probes (t-test, Benjamini-Hochberg-adjusted, p<0.05) between any combination of the four asthma k-means clusters (Asthma-1 to -4 (red, green, blue and pink circles, respectively)). The percent variation explained by PC1 and PC2 is given on the axis label. (**B**) Exacerbation-free rate for Asthma-1 and Asthma-2 subjects. (**C**) Results from IPA upstream modulator analysis for asthma Proteins-A to -F. Shown are the top 5 upstream modulators enriched in Proteins-A (top left), Proteins-B (top middle), Proteins-C (top right), Proteins-D (bottom left), Proteins-E (bottom middle), Proteins-F (bottom right).

**Figure S9. The three distinct COPD endotypes major proteins associate with Kco %predicted and immune cells**

(**A**) PCA scatterplot across all COPD subjects and the 121 significantly different probes (t-test, Benjamini-Hochberg-correction, p<0.05) between any combination of the three COPD k-means clusters (COPD1 to -3 (olive green, teal, and purple circles, respectively)). The percent variation explained by each PC1 and PC2 is given on the axis label. (**B**) Distributions of correlations between protein abundance and baseline Kco %predicted in COPD Proteins-G and -H and all other proteins (other). Correlations are given as linear regression absolute R-values. (**C**) Scatterplot showing the correlation between baseline Kco %predicted and GDI2 protein abundance, for Asthma-1 to -4 (red, green, blue, and pink squares, respectively) and COPD-1 to -3 (olive green, teal, and purple circles, respectively). The R value for the correlation and its associated p-value is given.

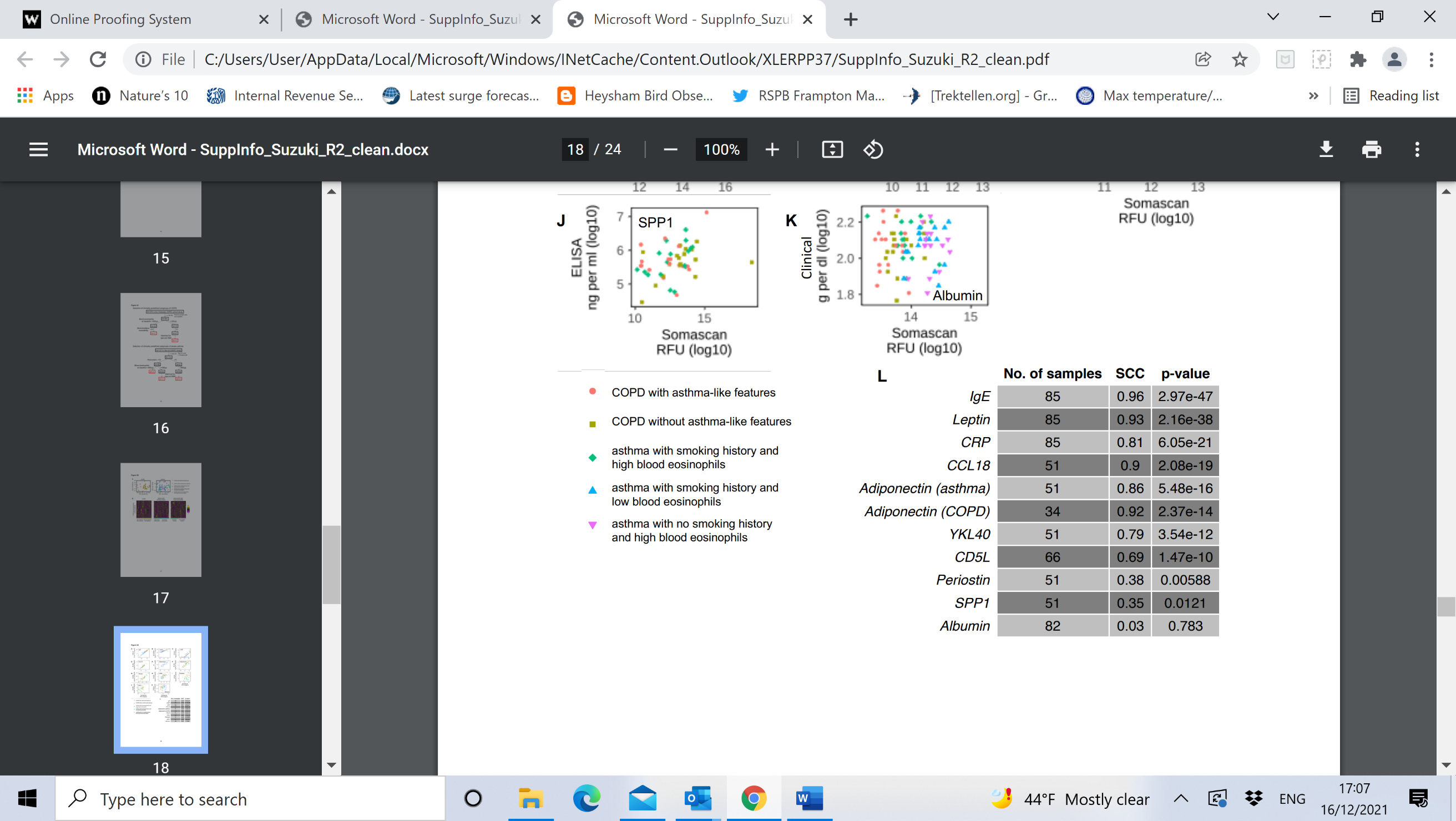

Supplement: Supplementary file 1 — Supporting Information S1 [file CLT2-11-e12091-s001.docx]
